# Facing Food Risk Perception: Influences of Confinement by SARS-CoV-2 Pandemic in Young Population

**DOI:** 10.3390/foods11050662

**Published:** 2022-02-24

**Authors:** Fernando Cantalapiedra, Cristina Juan, Ana Juan-García

**Affiliations:** Laboratory of Food Chemistry and Toxicology, Faculty of Pharmacy, University of Valencia, Av. Vicent Andrés Estellés s/n, Burjassot, 46100 València, Spain; fernando.cantalapiedra@uv.es (F.C.); ana.juan@uv.es (A.J.-G.)

**Keywords:** young population, food perception, risk perception, food safety, COVID-19

## Abstract

A new food safety level of trust in food risk perception has been noticed, as a consequence of the SARS-CoV-2 pandemic. The pandemic made-up to review nutritional recommendations for the population, mainly for the young population. Here, the results of a designed survey for the young population, from the University of Valencia, Spain, belonging to grades in the health branch of knowledge, and in charge of carrying out the shopping task for their household, are reported. The study reports three different scenarios and years, as defined by the SARS-CoV-2 pandemic: before the pandemic (period January–December 2019), during the pandemic lockdown (period March 2020–August 2020), and after the pandemic lockdown (September 2020–June 2021). The survey was designed with questions, profiling responses using the best–worst elicitation (BWE) format. Results reported that trust and evaluation of information differed in all three scenarios. In the SARS-CoV-2 pandemic, there was (i) a high increase in trust in the information provided *inside* (by) the shopping place, while there were no changes for the *outside* (kept in medium score); (ii) trust in cooperative stakeholders went from a medium-low to medium-high score, while, for individual stakeholders, it was maintained as a medium score, and (iii) trust in information on food products was kept in high score. Regarding the evaluation of the information provided by stakeholders, a tendency in medium score was maintained, while that from the channels of distribution went from medium-low to medium-high for buying on-site. A uniform tendency was observed for online/other distribution channels for all three years and descriptors studied: “Internet”, “Farmer on-demand”, and “Cooperative consumers” (<50%). This research provides findings of implications that contribute to changing the perception of food risk, due to the COVID-19 pandemic, i.e., the adaptation of the young population, trust in safety and quality, and importance of coordination from all communication points to avoid negative or strongest consequences, in case of future lockdowns or health crisis.

## 1. Introduction

Food safety, in a globalized world, is a major concern in the food supply chain for consumers. The concept of food safety seems to be completed, when there is no food risk or perceived, while the evaluation of risk perception implies the trust in purchasing a product and behavior [1,2]. In this sense, two points can be considered of great importance in food safety: (i) trust in food products and (ii) the levels of communications, which vary depending on the country or cultural contexts. Both points influence food perception across countries [3]. There are plenty of stages, indirectly implicit in the acquisition, for consumers to choose and consume a food product, which makes it necessary to give a wider trust in food, for the population, that guarantees food safety.

Food has several factors and connotations, when spreading risk messages, related to culture, symbolism, family, and even religion; in fact, there is evidence suggesting differences between food and non-food risks [4]. There are risks and benefits associated with food, such as the presence of contaminants and nutritional contribution. From both perspectives, when there is an intention of spreading this information, it is necessary for all to be considered as communication strategies [5].

Literature reports that the response of consumers to food safety or confidence is different, depending on the risk origin [6,7], so that, if a different behavior of potential risks is subsequently adopted, if it is referred to as technological or natural risks [8,9]. Similarly, this happens in the type of exposure to such risk: chronic or acute. In the acute context, the natural risk might increase the risk perception; while, in chronic context, the information provided in the risk assessment process by authorities is available for specific groups of vulnerable populations, so that the relationship among different circumstances causes different behavior and perception of risks [10].

During COVID-19, adequate nutrition was correlated with several indicators that influenced nutritional education (family member at home, watching TV during mealtime, country, maternal education, etc.) [11]. It pointed out that it was necessary to review and reshape nutritional recommendations for the young population, due to the different nutrition behavior reported during the COVID-19 lockdown [11].

Besides the aspect commented, it is of great importance to highlight the globalization that the market has developed in the last years. Warranties of good functioning in globalization can be supported, if there is trust in the food supply chain at different levels: general (understood as an interpersonal trust), stakeholders on the food chain, food authorities, and food products [12]. Nowadays, there has been a new level of trust, as a consequence of the SARS-CoV-2 pandemic, which is reflected in the study, presented here, for the young population. In this sense, the main focus of this study was to evaluate how the young population was facing food risk perception before, during, and after the lockdown pandemic, as well as what the most influential source/guide that provided safety and quality was.

National institutes of statistics have had to face the new scenario with COVID-19 by working on guidelines to obtain new methodologies of generating statistics, but most importantly to continue providing help in obtaining data across the globe [12,13,14,15,16]. One of the first steps in communicating food risk is to understand how consumers perceive that risk, and this is supported by involving science, evidence, and data regarding risk analysis; nevertheless, decisions in risk are also sustained by instinctual and/or emotional factors. These aspects have been indicated in previous studies; however, in the last year (referred to 2021), after the SARS-CoV-2 pandemic situation (after strict lockdowns), it could affect this perception and denote an increase in the demands of safety and quality on the part of the consumer and consumer agencies. Hence, the results of a designed survey for the young population from the University of Valencia, Spain, belonging to grades in the branch of health sciences and in charge of carrying out the shopping task for their household, are here reported (Table 1). The study collects the trust, regarding different sources of information, that gives warranties in the food chain, as well as how it is perceived for the young population. The study reports three different scenarios, defined by the SARS-CoV-2 pandemic: before the pandemic (period January–December 2019 “*normal life*”), during the pandemic lockdown (period March 2020–August 2020 “*during lockdown*”), and after the pandemic lockdown (September 2020–June 2021 “*after lockdown*”).

## 2. Material and Methods

### 2.1. Respondents

**Figure 1:**
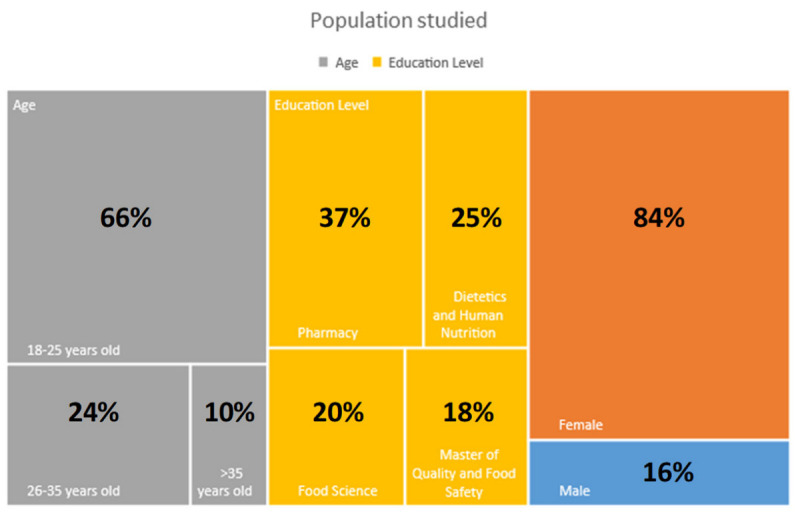
Characteristics of the young population studied, according to age, education level, and gender.

Data were collected in Spain from students of the University of Valencia, through a validated survey, from 2019 to 2021 (Ethics Committee at University of Valencia—UV-INV-1942475). The sample, consisting of 600 individuals, represents the young population, who are in charge of shopping baskets for the household (Table 1). The information collects responses of students from different health branch degrees: human nutrition and dietetics, pharmacy, food science, and masters’ degrees in quality and food safety. Students were contacted in a class by leaving the survey design open, with an age range from 18 to >35 years old. Characteristics of the population studied are collected in Table 1 and Figure 1. The survey was open for answering, for the groups described above; 84% were female respondents, and 16% were male. The order of respondents, per educational level, was pharmacy degrees, followed by dietetics and human nutrition, food science, and quality and food safety master’s degrees (more details of enlisted respondents are in Section 3).

### 2.2. Survey Design and Recruitment

**Figure 2:**
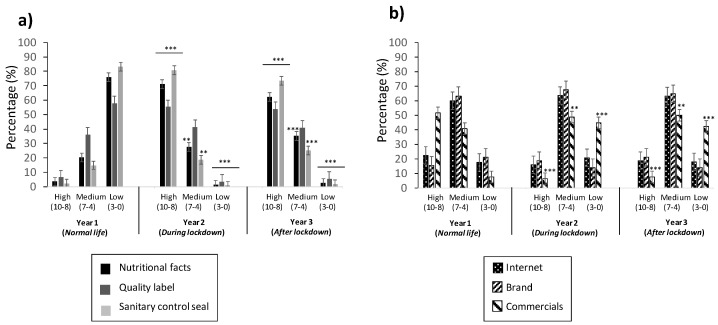
Trust of young population in information source of food products inside the shopping place (**a**) (“nutritional facts”, “quality label”, and “sanitary control seal”) and outside the shopping place (**b**) (“internet”, “brand”, and “commercials”). Values correspond to the mean ± SD of population responding to the questionnaire. ** p ≤ 0.01 and *** p ≤ 0.001, with respect to the control (Year 1).

**Figure 3:**
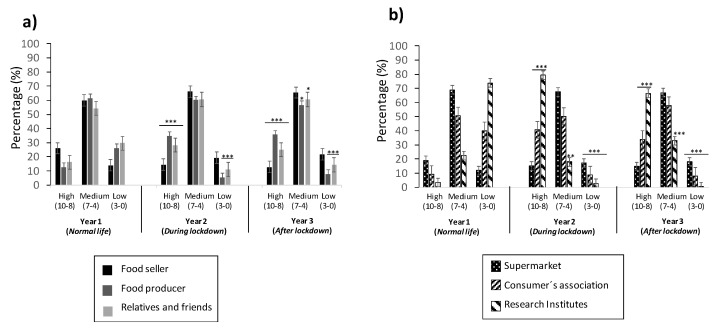
Percentage of trust of young population in stakeholders involved in the food chain production: individual (“Food seller”, “Food producer”, and “Relatives and friends”) (**a**) and cooperative (“Supermarket”, “Consumer’s association”, and “Research institutes”) (**b**). Values correspond to the mean ± SD of the population responding the questionnaire. * p ≤ 0.05, ** p ≤ 0.01, and *** p ≤ 0.001, with respect to the control (Year 1).

**Figure 4:**
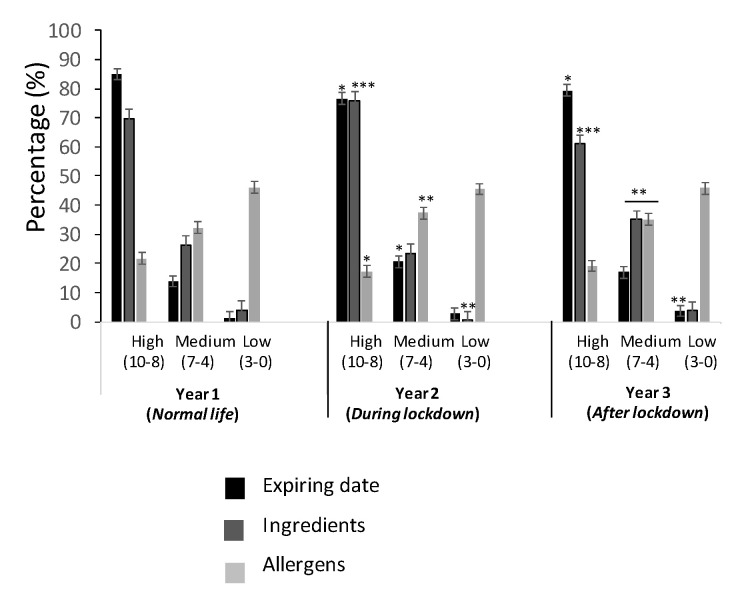
Percentage of young population checking information in food products, referred to: “Expiring date”, “Ingredients”, and “Allergens”. Values correspond to the mean ± SD of the population responding to the questionnaire. * *p* ≤ 0.05, ** *p* ≤ 0.01, and *** *p* ≤ 0.001, with respect to the control (Year 1).

**Figure 5:**
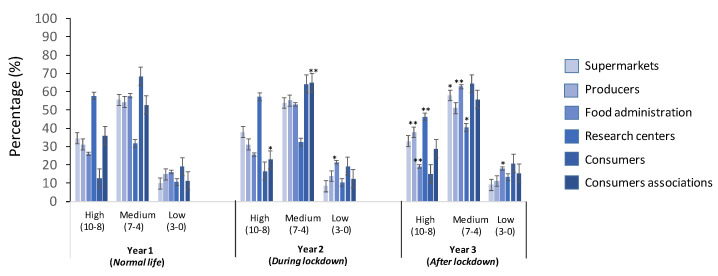
Evaluation of stakeholder in providing information of food products for the young population (“Supermarkets”, “Producers”, “Food administration”, “Research centers”, “Consumers”, and “Consumers associations”). Values correspond to the mean ± SD of the population responding to the questionnaire. * *p* ≤ 0.05, ** *p* ≤ 0.01, with respect to the control (Year 1).

**Figure 6:**
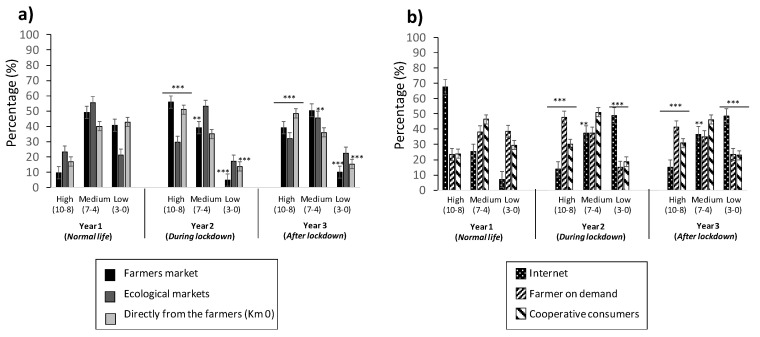
Evaluation of different channels for the young population to buy food: (**a**) on-site (“farmers market”, “ecological markets”, and “directly from the market”); (**b**) online/others (“internet”, “farmer on demand”, and “cooperative consumers”). Values correspond to the mean ± SD of the population responding to the questionnaire. ** *p* ≤ 0.01, and *** *p* ≤ 0.001, with respect to the control (Year 1).

A survey was designed, with questions profiling responses using the best–worst elicitation (BWE) format (described in Petrolia et al., 2016 [17]). The BWE format refers to getting answers that indicate only the first-best choice, and it has been used in several studies, as reported in the literature [18,19,20,21,22,23]. It describes, somehow, an order of what is the best alternative, followed by the worst and those remaining, again, the “best” and worst, until all options are ranked. The methodology results are easier to obtain than the standard question format, and it also permits our group to understand the best–worst ranked answers for the risk perception of the population studied. The structure of the questionnaire corresponded to three sections: trust in those providing information of food (source and stakeholders of the food chain) (Figure 2 and Figure 3), frequency of consumers in checking the information present in food (Figure 4), and evaluation of the information provided (Figure 5 and Figure 6).

### 2.3. Questionnaire Used for the Survey

The questionnaire was developed based on “best-worst elicitation” (BWE), in order to analyze whether the pandemic, associated with SARS-CoV-2 attributes, was associated with food safety and risk perception. The complete initial questionnaire consisted of 17 questions, previously tested through a pilot study, for its validation by the Organization of Consumers and Users—OCU organism in 2017 [24]. The questions selected for the survey were based on the existing data in the literature, carried out as a preceded task, while the discussed and reported results are based on those that gave a greater relevance. A scale from 0 to 10 points was used to describe the profile of trust or information from stakeholders, and the following score-levels were defined: (i) low: from 0 to 3 points; (ii) medium: from 4 to 7 points, and (iii) high: from 8 to 10 points. The questionnaire was answered in three different periods, as defined by the SARS-CoV-2 pandemic: before pandemic (*normal life*—Year 1), during pandemic lockdown (Year 2), and after pandemic lockdown (*end of lockdown*—Year 3).

### 2.4. Statistical Analysis

BWE allowed us to know the number of times an attribute of COVID-19 was selected as the most (best) or least influential (worst), as well as the average score for each attribute, for the entire sample, which allowed us to build the different figures described in the Results sections. Statistical analysis of data was carried out using the IBM SPSS Statistic version 23.0 (SPSS, Chicago, IL, USA) statistical software package and GraphPad Prism 8.0 (GraphPad Software, Inc., San Diego, CA, USA). Data were expressed as mean ± SD of three independent experiments. The statistical analysis of the results was performed by student’s *t*-test for paired samples. Differences between groups were analyzed statistically with ANOVA, followed by the Tukey HDS post hoc test for multiple comparisons. The level of *p* ≤ 0.05 was considered statistically significant.

## 3. Results

### 3.1. Best–Worst Scores and Respondents

The best–worst methodology allowed us to identify the most influential COVID-19 attributes, as considered by food companies during the pandemic. It was contemplated to have the different informative risk perceptions from each respondent that would influence their choices. Respondents were asked to provide a numerical rating for what they perceived to be the level of food safety for food, in general, without specifications but with a background on their studies (following BWE, described in Section 2.2). Results could change in populations with non-informative risk perceptions. Scores were divided into low, medium, and high categories, according to the description in Section 2.3.

The respondents’ profiles are reported in Table 1 and Figure 1. The total number of respondents was 600 students, corresponding to 16% males and 84% females; ages were between 18 to >35 years old, while the distribution by educational level was as follows: 37% studying for a pharmacy degree, 20% studying for a food science degree, and 25% studying for a dietetics and human nutrition degree. Finally, the population studying for the master’s in quality and food safety corresponded to 18%.

### 3.2. Trust in the Information Source

The information source in the young population revealed a markedly different score of trust, according to the survey, which is reported in Figure 2. It was divided by the information perceived (i) in the market, referred to as before or during purchasing (*inside* the shopping place) and contained on the packaging label (nutritional facts, quality seal, and sanitary control seal) (Figure 2a), and (ii) external (*outside*) to the shopping place, referred to as information not printed on the label but perceived from media (internet, brand, and commercials) (Figure 2b).

Figure 2a reports that, before COVID-19 “*normal live*” (Year 1), the trust of information contained on the label (*inside* the shopping place) was low for all three descriptors (nutritional facts, quality seal, and sanitary control seal), ranging from 58% to 83%, corresponding to “Quality seal” and “Sanitary control seal”, respectively. This tendency changes drastically during and after COVID-19’s lockdown (Year 2 and 3), as it went to high trust for all the three descriptors, above mentioned, with percentages of trust similar to those reported in low trust, from 54% to 73%, for the same descriptors as in Year 1 (Figure 2a). The medium trust for all three years studied revealed the following order of descriptors: “Quality seal” (from 36% to 41%) > “Nutritional facts” (from 20% to 35%) > “Sanitary control seal” (from 15% to 25%) (Figure 2a).

The results regarding the information perceived from “outside” of the shopping place, and mostly through media, is reported in Figure 2b. It reveals that the trust for the young population is in the medium score for the three descriptors (internet, brand, and commercials) and three years studied. The order that followed was: “Brand” (from 63% to 68%), “Internet” (from 60% to 62%), and “Commercials” (from 41% to 50%) (Figure 2b). When observing the “Commercial” factor, an interesting behavior, in all three years studied, for the high score of trust, was observed, as it went from 50% to 7% for Year 1 to Years 2 and 3, respectively; while, inversely, behavior was observed for the low score of trust, as it went from 8% to 45% for Year 1 to Years 2 and 3, respectively (Figure 2b). The “Internet” and “Brand” factors were maintained very similarly in all three years, for both high and low trust, in the ranges from 15% to 23% and 14% to 21%, respectively.

### 3.3. Trust in Stakeholders in Food Chain Production

Figure 3 reports the trust of the young population in the stakeholders involved in the food chain production, divided into individuals (Figure 3a) and cooperatives (Figure 3b). Trust by “Individuals” was maintained in the medium score for all three descriptors (sellers, producers, and relatives and friends), and the three years studied in the highest values, ranging from 54% to 61%. When observing the high score, increases of trust were observed for the (i) “Producer” descriptor, ranging from 13% to 36% for Years 1 to 3, respectively, and (ii) “Family and Friends” descriptor, ranging from 16% to 28% for Years 1 to 2, respectively, while decreases were observed for the “Seller” descriptor, ranging from 26% to 13% for Years 1 to 3 (Figure 3a). Results opposite to this were observed in the low score, as a decrease of trust values was observed for the (i) “Producer” descriptor, ranging from 26% to 8% for Years 1 to 3, respectively, and (ii) “Relatives and Friends” descriptor, ranging from 30% to 11% for Years 1 to 2, respectively, while an increase was observed for the “Seller” descriptor, ranging from 14% to 22%, from Years 1 to 3 (Figure 3a).

Trust by “Cooperatives” includes three descriptors: supermarket, consumer´s association, and research institutes. Results revealed that “Supermarkets” had similar values in all three years, from 12% to 69%, 15% to 68%, and 15% to 67% for Years 1, 2, and 3, respectively (Figure 3b). Notice that the upper bond values corresponded to the medium score. “Consumer´s association” provided a change in the values of trust from Years 1 to 2, as it went from a low to high score of trust (40% in both cases), although the highest values were for the medium score (50%). In Year 3, the percentage of trust was maintained, as well as for Year 2, although an increase of 8% was reported for the medium score, with a decrease of 8% for high score. Regarding the factor of “Research Center”, it was the factor that suffered the major variation, as it went from low score of trust (with 74%) in Year 1 to a decrease of 3% and 1% for Years 2 and 3, respectively; in consequence, the high score of trust went from 4% in Year 1 to 79% in Year 2, reaching 66% in Year 3 (Figure 3b).

### 3.4. Checking Information in Food Products

Figure 4 reports the results related to checking the information in food products at the moment of buying, referred to as the “Expiring date”, “Ingredients”, and “Allergens”. The highest values of trust were reported for the high score for “Expiring date”, ranging from 77% to 85%, followed by “Ingredients” (from 61% to 76%). Notice that this behavior was maintained equally for all three years, with the following order of trust: high > medium > low. For “Allergens”, the highest values were reported for a low score, with 46% in all three years, followed by a medium score, ranging from 32% to 35%, and, finally, a high score from 17% to 22%. In summary, “Ingredients” and “Expiring date” are highly checked and were maintained practically equally in all three of the years studied, while “Allergens” maintained low values, without changing in all three years.

### 3.5. Evaluating Information from Stakeholders

The results of evaluating the information of the food products provided from stakeholders, such as “Supermarkets”, Producers”, “Food Administration”, “Research Centers”, “Consumers”, and “Consumers Associations”, are reported in Figure 5.

In general terms, it is noticed that the evaluation is maintained in the medium score for all three years, followed by high and low score in the last position. In detail, the medium score ranged from 32% to 68%, 32% to 65%, and 40% to 64% for Years 1, 2, and 3, respectively; the “Consumers” factor had the highest score, and the “Research Centers” factor had the lowest score. The high score of the evaluation ranged from 13% to 58%, 17% to 57%, and 15% to 46% for Years 1, 2, and 3, respectively; the “Research Centers” factor had the highest score, and the “Consumers” factor had the lowest score. Finally, the low score was scored with the lowest percentages, as follows: from 10% to 19%, 8% to 21%, and from 9% to 21% for Years 1, 2, and 3, respectively; the “Food Administration” factor had the highest score, and the “Supermarkets” factor had the lowest score. In summary, the medium score was reported for “Supermarket” and “Food administrations”, while high score was reached for “Research centers”.

### 3.6. Evaluating Channels for Purchasing Food Products

A predisposition for purchasing food products by using different channels of distribution, such as “Farmer’s market”, “Ecological markets”, “Directly from the market”, “Internet”, “Farmers on-demand”, and “Cooperative consumers”, was evaluated. Figure 6 is divided by channels that require us to move to a specific marketplace “on-site” (Figure 6a) and channels that allow for purchasing food products online (Figure 6b).

The tendency observed in Figure 6a was very similar for all three years, with evaluations fitting the medium scores from 40% to 56%, 35% to 53%, and 36% to 51% for Years 1, 2, and 3, respectively. In Year 2, the evaluation was very close to the medium score, with percentages ranging from 30% to 56%, due to the “Farmer’s market” and “Directly from the market” factors; however, in Year 3, there was an increase in the evaluation of the low score for all three factors, studied from 10% to 22%.

The results reported in Figure 6b reveal that “Internet” was the factor experiencing the strongest changes among all three of the years studied. It went from a high score evaluation of 68% in Year 1 to 15% in Year 3, subsequently reaching a low score in Year 3, with 48%. “Farmers on-demand” had a maximum medium score in Year 1 (38%), which was similar in Years 2 (37%) and 3 (35%). Similarly, this happened for “Cooperative consumers”, with medium score values of 46%, 51%, and 46% for Years 1, 2, and 3, respectively.

## 4. Discussion

Information sources in risk communication have a principal role in spreading the voice when facing and engaging food safety. According to several studies [25,26], the inclusion of new technologies (mainly through apps) has become the main source checked by consumers, with a special focus on mass media, as the main contributor, but also providing education to the population, allegedly due to the accurate reports [27]. However, the uncontrollable impact of such sources can be considered a negative contribution that emphasizes risks [28]. It has been demonstrated that behavior and decisions in food products are shaped by the consumer´s perception, contained in the information source [29]. Results, reported in Figure 2b, confirm the relevance of this fact, showing that trust in the information perceived by studied young population from a source “*outside*” the shopping place is highly distributed, rather than that from “*inside”* the shopping place, referring to that which is printed on the label (Figure 2a). The influence of this information source changed during and after the pandemic lockdown (Years 2 and 3 of study) by trusting more of the information contained on labels (Figure 2a). This observed behavior could be associated with an uncertain situation at the beginning of the pandemic, as news (and mass media) in the first lockdown reported the possibility of the virus spreading in food: “food could contribute and contain the virus SARS-CoV-2”, another fact that could justify the changes observed in the population target. While sources evaluated in Figure 2a could be perceived as closer to our target population (students with a background in food safety), the ones in Figure 2b are closer to a broader audience, no matter the studies or background. These results coincide with an extensive study carried out in the UK population [30], Netherlands [31], and USA (Texas) [32]. It also puts in manifestation that the target audience is a factor to consider in food risk communication and, indeed, food risk perception.

In a study carried out for the Italian population, the quality of a food product and reason for purchasing such products have been associated [33,34]. Similarly, this happens with the typical products or PDO-certified products related to a brand [33,34]. Quality brands and certifications of origin are indicators that make it easier for consumers to judge and strengthen the perception of quality; in fact, not looking for information on the label can increase uncertainty, regarding the ability to appraise the quality, and encourage a tendency to rely on certification [33]. Sometimes this quality is reinforced with seals of quality or sanitary control or details in the nutritional facts, all contained in the label of the food and/or food product. All three indicators are reported in Figure 2a, which had been highly trusted during and after the lockdown pandemic, Years 2 and 3, respectively.

When adding value to a food product, it brings an increase of quality in consumers´ perception. That value can be provided in different ways, as reported by Mascarello et al. (2015) [33], with a coordinated flow within the food product, based on creating, maintaining, and enhancing characteristics in the food product [33]. When providing these advantages, the information contained on labels is crucial to communicate them to the consumer; the broader the audience, the greater the role of labels, quality seals, and communication. Communication is the tool used by institutions that look into scientific evidence and focus on specific groups that define perception and target actions that help to promote healthy behaviors [35].

Additionally, the lifestyle, household composition, age of residents, and employment of a determined local area or population group affect the determination of quality and food risk perception [33]. However, when a scandal involves the food industry, only brands are associated with a guarantee of food safety, which sometimes can also fall into distrust [33,36,37]. This point is important, as it has been demonstrated that COVID-19 has a human-to-human transmission, which causes a complete, indirect effect on the food industry [38].

When focusing outside the shopping place, in a study carried out in Chinese population (aged 40 years old), regarding the information source reported by different channels, it was observed that television (TV) was the most-used channel, followed by the internet and “other sources” [39]. In Turkey, similar results were obtained, with TV and mass media as the main information sources of trust; however, government publications were highly trusted, which reinforced the point of helping to educate consumers by food authorities [40]. All this can vary, when focused on a specific type of food, as demonstrated in a study in population from South Korea, regarding genetically modified food (GMO); when exploring risk perception, in general, journalists and science journalists’ were the latest that had more trust [41].

Facing food safety during COVID-19, by the food industry, sparked special attention, and several studies started to come up describing or reporting the issue that had to be strengthened [42]. In a survey study, carried out in 16 countries and by more than 800 food companies, it was revealed that the most important attributes faced were the staff awareness and the implementation of restrictive hygiene procedures, following the two main documents that WHO had developed for the food supply chain [38,43]. The industry was not compromised at any moment, regardless of food safety, despite not having any protocol or emergency plan for a previous pandemic [42]. Since then, protocols of the food chain have been reinforced, emphasizing the hygiene of hands, disinfecting packages, use of adequate equipment of protection, and preparation of food [44].

The stakeholders implicated in food chain production, in providing information, supposes a factor that can alter the risk perception in the trust that the population puts in the information source, so that there are perceptions of the participants playing a determinant role, according to stakeholders in the food chain. In general, it is expected to have greater trust in those perceived as more knowledgeable, honest, or closer to the information, as demonstrated in a study carried out in different populations [35,39,45]; nevertheless, there are country cultural factors that influence such responses. Similar to the results obtained here, the punctuation obtained in this study can be related to the stakeholders involved in the food chain production, either individually or from cooperatives. In the study of Liu et al., 2014 [39], the factors “Food producer” and “Relatives and friends” were perceived with honesty and concern, respectively, for citizen´s health perception. In our study, both were perceived with the highest values, especially during the lockdown pandemic (Year 2), and classified as individual stakeholders involved in food chain production (Figure 3a). Among that, the factors “Consumer´s association” and “Research institutes” were perceived as honesty in providing accurate information [39]; in our study, the percentage of trust varied indistinctly for each year studied but were classified as cooperative stakeholders involved in food chain production (Figure 3b).

Consumers´ behaviors and attitudes toward safe food should be taken into account, in regard to perceived food safety, i.e., checking the package information of the food before purchasing, for example, the content of allergens, ingredients, expiring date, origin, calories, nutrition facts, and brand were measured in this study (Figure 4). One of the observations before purchasing a food product is the expiration date, referred to as the last date that a food should be eaten or used, i.e., understanding that, after that date, the characteristics of the food product are altered, and risk might occur. In a study carried out with the Turkish population, it was revealed that there is a rejection behavior to expiring date information [40]. In our study, this information was highly checked before pandemic (Year 1) and decreased during lockdown (Year 2) and after it (Year 3) (Figure 4). There was not further investigation carried out here to explain this, but it could be hypothesized that there was a high trust in food products, due to the no association of infection through them, despite the initial message from some news sources. EFSA declared that there was no scientific evidence that food was a risk or transmission route of the virus [46]. Publicity on TV and media were providing security in all food products, related to SARS-CoV-2, after a few months of the pandemic lockdown. Another issue reported in this study, as well as in Figure 4, is checking the information from the ingredients, which reported a similar behavior as that for expiring date; for allergens, this inversely correlated behavior, as compared to the expiring date and ingredients. The allergens content is information usually checked by consumers.

In evaluating the information provided by several stakeholders of the food production, in a study carried out with the Turkish population and referred to the information provided to them, it was evaluated as unreliable from that of scientists and specialists, while the government was evaluated with the highest value and responsibility to ensure food safety [40]. This does not coincide with our results, due to the different profile of population studied, not only in age but also in the country and studies background, which was the base of our population recruitment (Figure 5). Food manufacturers, scientists, and media were the greatest valuable stakeholders in providing product information, according to Rohr et al. (2005) [47], and even more trustworthy by consumers or environmental organizations [40]. This coincides with our results, as consumers were highly evaluated, and the highest values during the pandemic lockdown (Year 2). On the other side, it has been reported that nutritionists, which constitutes the group of the population studied here, have a high value in spreading information of food safety [40]; jointly, consumers´ association was extremely reliable. This fact was also observed here.

Finally, the preference for purchasing food products during the pandemic lockdown was also asked in our young population of study, as there was an increase of news in TV and media related to online (internet) shopping, coinciding with Brugarolas et al. (2020) [48], who also noticed a stockpiled food tendency during COVID-19, due to buying more often. That fact brought producers and distribution companies to develop strategies to decrease this effect or stock non-perishable foods. However, according to the group of the population studied here, there were no difference during the three years of study, in relation to the SARS-CoV-2 pandemic (Figure 6). The population was asked about their disposition to buying food products from different channels; besides the marked young age of the population studied, there was a high tendency of buying in markets of proximity, but also on the internet (online). Results are very hopeful, considering the new tendencies around food products and market introduced for new styles of life. In the study of [48], the population studied was broader (from 18 to more than 65) than the one reported here (Table 1).

It is important to mention that this study was focused on the risk perception for a specific group of the population, which coincides with the pandemic lockdown, due to SARS-CoV-19, without paying attention to any specific product type. The interest lays in analyzing the topic of risk perception, associated with food safety, as a co-complex field, as well as defining the behavior of young consumers. Additionally, a very unique and specific circumstance is reported, as several factors shifted the behavior, while, for others, this was maintained; it would be interesting to analyze the approach for a specific type of food product.

## 5. Conclusions

Consumers’ education starts with a strong trust in the basis of the information that is provided by the different stakeholders involved in food production/manufacturers. The alterations in food risk perception are produced when a pandemic sprouted/arrived and changed trust and confidence in several aspects. This situation has shown a high reliance and trust with nutritional facts, quality labels, and sanitary control seals, after and during the pandemic lockdown, as well as an increase in the trust of the farmers’ market, farmers of demand, and internet shopping. Results of this study put, in evidence, the importance of trust, regarding the information spread for food risk perception for the young population, with background studies in the field of food safety, as well as the implications of the legislation for some labels and stakeholders, which could be more influential in some aspects. Questionnaires of food risk perception, per group of population, help to give us a better idea of the perception of food safety, as well as to make comparisons between groups of population and focus the campaigns of education in food production. Nevertheless, further studies of collaboration would be necessary to have a broader picture of more countries for this group of the population.

## Figures and Tables

**Table 1:** Characteristics of respondents, corresponding to the young population from the University of Valencia.

Students	%
*Gender* Male Female	16 84
*Age* 18–25 years old 26–35 years old >35 years old	66 24 10
*Education Level Degree in* Pharmacy Food Science Dietetics and Human Nutrition Master of Quality and Food Safety	37 20 25 18

## Data Availability

Not applicable.
